# Three-dimensional microelectrode arrays for *in vitro* neuronal network models: structural designs, manufacturing approaches, and future perspectives

**DOI:** 10.1007/s13534-026-00610-y

**Published:** 2026-08-12

**Authors:** Dongjo Yoon, Yoonkey Nam

**Affiliations:** 1 Department of Bio and Brain Engineering, Korea Advanced Institute of Science and Technology (KAIST), Daejeon, Republic of Korea; 2 Information and Electronics Research Institute, Korea Advanced Institute of Science and Technology (KAIST), Daejeon, Republic of Korea

## Abstract

Three-dimensional (3D) microelectrode arrays (MEAs) have been actively developed to enable electrophysiological recording from *in vitro* 3D neuronal network models, which have attracted considerable research interest in recent years. These devices overcome the fundamental limitation of conventional planar MEAs, in which microelectrodes are confined to a single plane, by distributing microelectrodes throughout 3D space. This enables direct access to neurons throughout the tissue, allowing precise analysis of neural activity in 3D space across the neuronal network. Depending on the strategy used to position microelectrodes in 3D space, current 3D MEAs have been developed in a variety of structural configurations, including probe-, tip-, planar mesh-, 3D mesh-, curved surface-, stretchable mesh-, and wrapping-architectures. These structurally diverse devices have primarily been fabricated using photolithography-based microfabrication processes, while more recently, 3D printing technology has been adopted to improve structural design freedom for complex 3D structures. In this review, we discuss the structural diversity and fabrication strategies of 3D MEAs for *in vitro* 3D neuronal network models, examine recent advances and current limitations, and outline future directions for the field.

## Introduction

An *in vitro* neuronal network model is an experimental platform comprising neuron networks cultured under laboratory conditions, designed to investigate complex brain systems in a precise and controlled manner. These models have been widely used to study structural and functional neurophysiological phenomena of the nervous system, including the electrophysiological properties of network-level functional interactions [1, 2]. Across the field of tissue engineering, three-dimensional (3D) tissue models have been increasingly reported to more faithfully mimic the mechanical properties and biochemical microenvironment of tissue compared to conventional 2D culture systems [3, 4]. This trend has extended to neural system modeling, generating considerable interest in 3D neuronal network models in which neurons form connections and interact within a 3D space [5]. Indeed, 3D neuronal networks have been reported to exhibit more active spike firing [6], broader repertoires of activity patterns [7], diverse synchronization states [8], larger bursts [9], and more enriched gene expression profiles associated with neural development [10], compared to conventional 2D neuronal networks. Furthermore, the significance of 3D neuronal network models has grown alongside the rapid expansion of brain organoid research, which recapitulates the cellular complexity and self-organization underlying brain development and disease [5, 11]. Concurrently, methodologies for constructing 3D neuronal network models have diversified considerably. Current approaches include the culture of dissociated neurons within scaffolds such as silk [12–14], bead-based matrices [7, 15, 16], ECM-derived hydrogels [10, 17–20], and 3D printed scaffold [21], as well as the formation of spheroids and organoids through self-assembly [5, 11, 22]. Consequently, contemporary *in vitro* 3D neuronal network models encompass a variety of forms including spherical [23, 24], cubic [18, 20, 25, 26], doughnut [20, 26], lattice [27–29], cylinder [12, 13, 30–32], assembled spherical [33–35], and complex configurations [36, 37], with sizes ranging from hundreds of micrometers to several millimeters. As interest in the electrophysiological activity generated by these 3D neuronal network models has grown, extensive research has been conducted on readout technologies for signal measurement.

Microelectrode array (MEA) platforms have been widely used to measure the electrophysiological activity of *in vitro* neuronal networks [38]. MEA systems enable long-term recording without additional biological manipulations, such as genetic modification. Owing to this advantage, they have been widely employed to study spontaneous activity in *in vitro* neuronal network models. Initial efforts to record neural activity from 3D neuronal network models relied on conventional planar MEAs, also known as two-dimensional (2D) MEAs [7, 9, 24, 26, 33, 39]. However, 2D MEA platforms are limited to recording neural signals only from the surface neurons in contact with microelectrodes confined to a single plane, which means they can capture only a fraction of the neurons distributed throughout 3D space. These limitations lead to insufficient sampling of neuron populations located within 3D neuronal networks, hindering the accurate interpretation of the spatial and functional structure of the entire network. To address this, 3D MEA technologies have been actively developed, in which microelectrodes are arranged in three dimensions to enable direct access to neurons at various positions within the 3D structure. Throughout this review, we define *true 3D recording* as the capability to electrically access and spatially distinguish neurons distributed across all three spatial axes of a tissue model, not only along the lateral (x–y) plane but also along the depth (z) axis, thereby enabling spatially resolved analysis of activity throughout the tissue volume rather than only at its surface or within a single plane.

Here we review 3D MEA technologies developed for electrophysiological recording from *in vitro* 3D neuronal network models, with a focus on structural characteristics, fabrication strategies, and recent technological advances. In addition, through a comparative analysis of the advantages and limitations of each technology, we discuss prospective directions for 3D MEA development and the key technical challenges that remain to be addressed.

## Various structures of 3D MEAs based on interfacing approaches

**Fig. 1:**
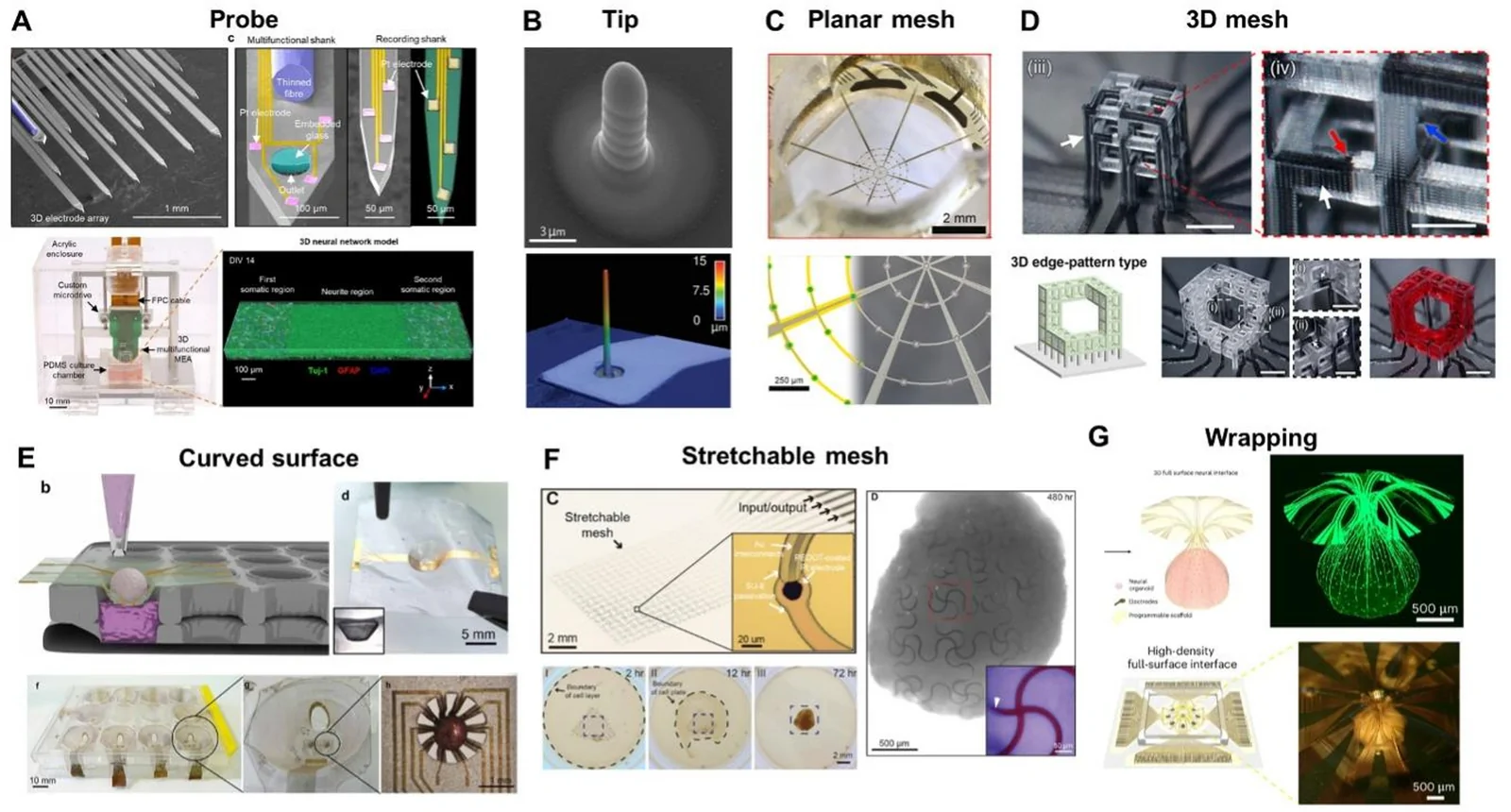
Diverse structure of 3D MEAs. **A** Probe architecture. Adapted with permission from [40]. Copyright 2021 Springer Nature. **B** Tip architecture. Adapted with permission from [41]. Copyright 2019 John Wiley and Sons. **C** Planar mesh architecture. Adapted with permission from [42]. Copyright 2023 Elsevier. **D** 3D mesh architecture. Adapted with permission from [20]. Copyright 2025 John Wiley and Sons. **E** Curved surface architecture. Adapted with permission from [43]. Copyright 2026 IOP Publishing. **F** Stretchable mesh architecture. Adapted with permission from [44]. Copyright 2019 American Chemical Society (ACS). **G** Wrapping architecture. Adapted with permission from [45]. Copyright 2026 Springer Nature

A 3D MEA for *in vitro* 3D neuronal network models is a device in which microelectrodes are arranged three-dimensionally within a small space, ranging from hundreds of micrometers to several millimeters. The structural design of these devices varies depending on how the microelectrodes physically access the 3D neuronal network model. Broadly, they can be classified into two categories: fixed 3D MEAs, in which electrode positions are fixed during interfacing, and compliant 3D MEAs, in which the device mechanically deforms to interface with the model (Fig. 1; Table 1).

### Fixed 3D MEA

A fixed 3D MEA is a device in which the microelectrode positions defined during fabrication remain stable upon interfacing with a 3D neuronal network model. This allows a direct mapping between electrode locations and recording sites, making it well-suited for spatial interpretation of the recorded signals.

#### Probe architectures

The probe architecture is one of the most representative forms of 3D MEA. Microelectrodes are arranged along a linear, rod-shaped structure known as a shank, which can be used as a single unit or integrated into multi-shank arrays to expand recording coverage [40, 46–49] (Fig. 1A).

A key feature of this architecture is its ability to be directly inserted into *in vitro* 3D tissue models, similar to its use in *in vivo* brain recordings. Probe-type 3D MEA has been applied to a wide range of models, including neuron-hydrogel composite models [40, 47, 50], brain slices [48], spheroids [51], and organoids [39, 52–57]. In particular, probe-type 3D MEAs offer the advantage of selective access to individual specimens at a desired interfacing time point, as well as depth-selective readout across varying insertion depths [58, 59]. More recently, bendable probe-type 3D MEAs have been reported that incorporate magnetically responsive materials, enabling post-insertion repositioning of microelectrodes within the model by bending probe segments via an external magnetic field [60]. This approach maintains the insertion capability and positional selectivity of conventional probe architectures, while allowing additional spatial adjustment after insertion.

However, insertable designs require physical penetration of the model, raising concerns about tissue damage and invasive interfacing. In densely packed models such as organoids and spheroids, insertion can cause mechanical disruption and compromise tissue integrity over long-term culture. To eliminate this damage, several studies have proposed a non-invasive approach involving the direct cultivation of neurons on the devices [40, 47]. Moreover, single-shank designs inherently limit microelectrode placement to a single plane [54, 55]. Thus, even with electrodes distributed along its length, recording remains restricted to the z-axis rather than achieving true 3D recording. This limitation persists even with multiple shanks if they are arranged along only one lateral direction with narrow inter-shank spacing. True 3D recording has therefore been achieved using two-dimensionally arranged, Michigan-probe type shank arrays distributed across both lateral axes (x and y), enabling electrode placement throughout the full 3D volume [40, 47, 50].

#### Tip architectures

The tip architecture extends the conventional planar MEA by fabricating microelectrodes with protruding structures, enabling signal acquisition from cells located in the 3D space above the substrate (Fig. 1B). Unlike conventional planar MEAs, tip electrodes can make contact with cells at intermediate depths within the 3D space. Tip-type microelectrodes are typically formed with small widths of 3–19 μm and heights of up to 190 μm [41, 61, 62], while larger variants have been reported with widths of 40–80 μm and heights of up to 250 μm [63–68], and widths of 150–320 μm and heights of up to 300–500 μm [69–71]. This has broadened the applicability of this architecture to neuron-hydrogel composite models [66], brain slices [63, 68], ex vivo tissue [64], and organoids [62]. An advantage of this design is its compatibility with existing planar MEA instrumentation, lowering the barrier to adoption for current MEA users.

However, as the uninsulated conductive structures often extend for tens to hundreds of micrometers, the spatial extent of signal acquisition may increase. This makes it difficult to precisely localize the origin of recorded signals, potentially reducing spatial resolution. Additionally, the high aspect ratio of these protruding electrode structures raises concerns about mechanical breakage during interfacing with the model.

#### Planar mesh architectures

The mesh architecture is based on the 2D MEA design, in which the microelectrodes and conductive lines are retained while the surrounding substrate material is removed to create an open-mesh structure (Fig. 1C). In this design, microelectrodes are arranged on a thin mesh frame, which becomes fused within the tissue as it grows [42, 72].

This approach was proposed to overcome the limitation of conventional 2D MEAs, which can only interface with the tissue surface. When the mesh is incorporated during the culture of self-assembling and self-organizing models such as organoids [42, 72], the microelectrodes can become partially embedded within the tissue as it develops. This enables recording of neural activity from neurons located inside the tissue.

However, the microelectrodes in mesh MEAs are often arranged on a single plane spanning only the lateral x-y axes. Consequently, these devices do not constitute true 3D recording, as they cannot independently resolve neural activity along the depth (z) axis. As a result, precise separation and recording of activity from neuronal populations broadly distributed along the z-axis remain structurally challenging.

#### 3D mesh architectures

To overcome the limitation of the planar mesh architecture, 3D mesh-type MEAs have been developed in which microelectrodes are distributed across all three spatial dimensions, thereby achieving true 3D recording in which neurons are directly accessed at multiple positions along all three spatial axes rather than only along a single plane. In this design, microelectrodes and conductive lines are formed along insulator-based mechanical supports within the 3D space, while the open regions between them allow tissue to grow and interconnect, ultimately enabling an interface in which electrodes are distributed throughout the interior of the tissue [20, 73–75] (Fig. 1D).

More recently, this 3D mesh structure has been explored beyond simple signal recording, serving as a scaffold for patterning the architecture of 3D neuronal network models [20]. By loading dissociated neuron-hydrogel mixtures into scaffolds of various geometries, including cubic, bridge-connected modular, and 3D doughnut structures, both the shape of the 3D neuronal network and the electrode configuration can be defined simultaneously. This approach enables precise 3D spatial analysis of neural activity across differently shaped networks through microelectrodes fixed at the core and outer regions of the tissue.

However, 3D mesh MEAs are not well-suited for interfacing with pre-formed, densely packed tissue models, as insertion of the mesh structure is highly invasive and can cause significant cellular damage. Therefore, this architecture is suited for culture approaches in which the formation of the tissue model and device integration occur simultaneously.

#### Curved surface architectures

The curved surface architecture features a substrate fabricated in a curved geometry to enable more stable contact with spherical tissue surfaces, such as organoids (Fig. 1E). This design addresses the limited contact area and poor positional stability encountered when spherical tissues are placed on 2D MEA substrates [43, 76, 77].

This architecture is well-suited as a post-culture readout platform, as fully developed organoids can be placed onto the curved substrate at any desired time point, enabling stable contact and secure positioning between the tissue surface and the microelectrodes. These devices feature 6 to 59 microelectrodes arranged on a single curved surface, enabling 3D electrophysiological analysis of tissue models. Integration with multi-well plates has further extended their utility toward high-throughput analysis of large numbers of organoids [43].

However, since electrodes contact only the tissue surface, electrophysiological signals from cells located deep inside the tissue cannot be directly recorded. Furthermore, to transfer the spherical tissue models, the curved surface-type MEA must be fabricated in a hemispherical form, limiting signal acquisition to only half of the model surface. To address this issue, a study has proposed using two curved MEAs to cover the entire surface of the tissue model [77].

### Compliant 3D MEA

While fixed 3D MEAs use fixed electrode positions defined during fabrication, compliant 3D MEAs use flexible electronics to achieve true 3D recording by physically deforming the device to bring microelectrodes into contact with 3D neuronal network models. In this approach, electrodes and conductive lines are first fabricated in a flat, planar form, then shaped into a 3D structure or fitted to the tissue surface through stretching or wrapping. Depending on which side, the device or the tissue model, actively drives this conformal interfacing, compliant 3D MEAs can be further distinguished into two architectures: the stretchable mesh architecture, in which the device passively deforms in response to tissue growth or an externally applied stretching force, and the wrapping architecture, in which the device itself actively folds or bends to enclose the tissue model.

#### Stretchable mesh architectures

The stretchable mesh architecture consists of a substrate carrying electrodes and conductive lines designed to be geometrically extensible. Using lattice, kirigami, or serpentine patterned ultrathin stretchable mesh electrodes, typically 3–20 μm in width and 0.5–2.8 μm in thickness, the planar device can stretch and conform to the tissue model structure. This architecture can interface with 3D neuronal network models in two main approaches. The first approach involves co-culturing the device with a 3D neuronal network model such as an organoid, allowing the mesh to become gradually embedded within the tissue model. The second approach involves bringing the device into surface contact with a fully developed organoid at a desired time point to record signals after culture. In both approaches, the device itself does not actively change its own shape. Instead, it passively follows the shape changes imposed by tissue growth or an externally applied stretching force.

In the first approach, the embedded stretchable mesh architecture is designed to be incorporated into developing tissue models from the early stages of formation and is naturally internalized as the tissue grows [44, 78–81] (Fig. 1F). This approach, which is often referred to as the cyborg organoid strategy, is characterized by the integration of electronic components at multiple locations within self-organizing organoid models [44, 79]. This enables long-term longitudinal monitoring of electrophysiological activity throughout brain organoid development, including electrically evoked responses and pharmacological responsiveness [78, 79, 81]. The low bending stiffness of the mesh also minimizes mechanical stress on the tissue, making it well-suited for long-term culture and chronic recording.

In the second approach, the surface-contacted stretchable mesh architecture can wrap under applied force, conformally adhering to curved 3D surfaces such as those of organoids. This architecture is well-suited for non-invasive readout of spherical 3D tissue models after culture [82–85]. It offers flexible adaptation to surface curvature and the ability to interface with multiple organoids simultaneously [82]. Additionally, the reduced mechanical mismatch compared to rigid shells minimizes tissue compression and damage, while the device can deform alongside growing organoids to maintain conformal contact. By using two stretchable mesh-type MEAs positioned on the upper and lower hemispheres, neural signals from the entire surface of the organoid could be recorded [84].

However, in the first approach, the electrodes are embedded and grown together with the cells during cultivation, so their final positions may change over time, making it difficult to precisely define and interpret the electrode coordinates. In the second approach, these surface-contacted stretchable mesh architectures are unable to directly access cells within the tissue interior. Also, the fabrication process is technically demanding [82], and scaling to high-throughput platforms for simultaneous analysis of multiple tissue models remains challenging. Moreover, structural deformation during use may shift the relative electrode positions, reducing spatial reproducibility compared to fixed 3D MEA designs.

#### Wrapping architectures

The wrapping architecture consists of a flexible electrode array fabricated in a flat form that bends or folds to wrap around a 3D tissue model (Fig. 1G). Using self-folding [86–91] or mechanically guided bending [45, 92], the electrode array is designed to fold inward and wrap around the tissue surface once the model is placed inside the device. Unlike the stretchable mesh architecture, in which the device passively conforms to tissue growth or an external stretching force, the wrapping architecture is defined by the device itself actively driving its own folding or bending motion to enclose the tissue model, independent of tissue growth. This architecture is particularly well-suited for spherical or ellipsoidal tissue models such as spheroids [87, 89, 90, 92], organoids [45, 86, 91], and assembloids [92], as it allows microelectrodes to contact the tissue surface from multiple directions, greatly improving surface recording coverage. Most MEAs based on this architecture maintain a uniform electrode distribution across the tissue surface. In a recently developed high-density MEA with 240 electrodes, electrode yields exceeding 90% were achieved, enabling detection of oscillatory waves across the entire recording area [45]. Furthermore, since the device wraps around the curved tissue surface without deep insertion, it reduces tissue damage compared to rigid probes while enabling stable multi-channel signal acquisition. The 2D fabrication process also facilitates high channel integration and relatively high electrode density, which are key advantages of this architecture.

However, since this architecture wraps around the exterior of the tissue, recorded signals are primarily limited to cells at the model surface. Additionally, the mechanical properties of the wrapping structure must be carefully designed to match the tissue model size, ensuring stable contact without causing tissue deformation [93]. Since tissue models vary in size and shape, applying a single wrapping structure to all models remains challenging.

##  Fabrication strategies for 3D MEAs

3D MEAs have been developed using a wide range of microfabrication techniques, which can be broadly categorized into two approaches: microfabrication process-based and 3D printing-based approaches (Table 1). The former uses well-established planar microfabrication techniques from the semiconductor industry to achieve precise electrode arrays and high electrical integration, while the latter allows greater structural design freedom by building 3D structures layer by layer through additive manufacturing.

### Microfabrication process-based approach

The microfabrication process-based approach is the most established fabrication method in MEA development, widely adopted for its high technical maturity, reproducibility, precise micropatterning, and potential for industrial scalability. Based on well-defined silicon microfabrication techniques such as thin-film deposition, etching, passivation, and metal patterning, this approach enables reliable and repeatable fabrication of microelectrodes, conductive lines, and insulating layers with high precision. It is particularly well-suited for achieving high-density electrode arrays and complex wiring structures within a limited area, which is critical for platforms requiring high spatial resolution, such as high-density MEAs [40, 45, 94, 95] and stretchable mesh-type MEAs with sub-micrometer substrate features [82, 83]. This approach also facilitates integration with transistor-based circuits, enabling the development of highly integrated systems such as CMOS MEAs [96]. Furthermore, the incorporation of microfluidic channels [97–99] and optical sources [40, 92, 100] into the fabrication process enables the development of multifunctional 3D MEAs, highlighting the broader scalability of this approach.

However, the microfabrication process is inherently limited by its planar fabrication processes. Since most fabrication steps involve layer-by-layer deposition and patterning on flat substrates, there are structural constraints on arranging microelectrodes throughout a true 3D space. Therefore, existing 3D MEAs have relied on post-processing techniques such as assembling planar structures into insertable probe arrays through multi-layering [40, 94]. More recently, research has actively combined mechanically flexible materials and structural deformation strategies with microfabrication-based fabrication. The use of soft materials such as SU-8 and polyimide allows planar microfabricated structures to acquire flexibility enabling devices initially fabricated in a 2D form to be conformally adapted to the surface or interior of 3D tissue models [86, 92, 101, 102]. This represents an important and growing trend in the field of 3D MEA development.

### 3D printing-based approach

The 3D printing-based approach fabricates 3D MEAs by directly forming the desired 3D structure through additive manufacturing. This approach leverages the high structural design freedom, customizability, and use of biocompatible materials offered by 3D printing technology for fabricating complex 3D structures [103, 104]. This approach allows the device structure and electrode arrangement to be customized for various *in vitro* 3D neuronal network models, including neuron-hydrogel constructs, spheroids, organoids, and assembloids, which range from hundreds of micrometers to several millimeters in size and vary widely in shape. It also enables rapid structural modification and prototyping through adjustments to the printing model files.

Two main strategies have been used to fabricate 3D MEAs using 3D printing. The first involves directly printing electrically conductive materials in three dimensions. Material extrusion-based direct printing allows liquid metal or gold to be printed into 3D electrode structures [48, 60, 67–106]. Inkjet printing with conductive polymer inks enables fabrication of tip-type electrodes with diameters below 40 μm and heights below 90 μm [41, 61, 65], while silver nanoparticle ink-based inkjet printing produces probe-type electrodes with diameters of 23 μm and heights under 1 mm [57]. Aerosol-jet printing with silver or gold nanoparticle-based inks can produce probe-type 3D MEA ranging from hundreds of micrometers to several millimeters in height [62, 107]. Additionally, microstructures fabricated using a stereolithography (SLA) 3D printer have been coated with electrically conductive materials such as gold or platinum to produce microelectrodes with features on the scale of tens to hundreds of micrometers [69, 108, 109]. This strategy allows direct formation of conductive structures and enables rapid prototyping of electrodes with various sizes, heights, and arrangements. Typically, the conductive structure is first fabricated, followed by coating with a passivation layer such as parylene-C through a chemical vapor deposition (CVD) process, and then laser opening of the electrode sites to complete the final 3D MEA. However, due to the nature of continuous material extrusion and laser opening processes, this approach has limitations when applied to complex structures where the printing nozzle or laser focus cannot easily reach.

The second strategy involves first forming a 3D hollow insulating structure using non-conductive photosensitive resin, then filling the internal microchannels with electrically conductive materials to define the electrodes and conductive lines [20, 49, 110]. This extends the concept of 3D printing-based 2D MEA fabrication [111, 112] into three dimensions. It offers the advantage of freely positioning electrode opening holes during the insulator formation stage, without the constraints of additional laser opening processes. For example, two-photon polymerization-based 3D printing can be used to print a hollow insulating structure on top of a 2D MEA, which is then filled by gold electroplating to produce thin probe-type electrodes with high aspect ratios [49]. DLP-based 3D printing has also been used to form hollow 3D insulating structures with internal microchannels, which are subsequently filled with electrically conductive inks such as graphite, PEDOT: PSS, or silver nanoparticle ink by capillary action [20]. This approach enables greater freedom in placing microelectrodes and conductive lines throughout 3D space, making it well-suited for fabricating complex millimeter-scale 3D mesh structures. Furthermore, such structures can serve as scaffolds for directly loading neuron-hydrogel mixtures and guiding the 3D structure of the tissue model, offering potential as integrated platforms for tissue engineering and bioelectronics.

However, conductive line integration resolution remains a limitation compared to microfabrication-based processes, and the minimum feature size and structural design complexity are subject to constraints depending on the choice of equipment and materials. Process standardization is also not yet fully mature, leading to performance variations arising from material shrinkage, printing errors, structural deformation, and post-processing conditions. Nevertheless, 3D printing is regarded as a powerful alternative to conventional microfabrication-based processes, as it allows electrode positions to be freely defined in 3D space and enables rapid prototyping of complex structures.

**Table 1:** Comparison of 3D MEA architectures in terms of structure, fabrication method, advantages, limitations, and applicable *in vitro* tissue models

Categorization	Architecture	Fabrication method	Advantages	Limitations	Applicable tissue models	Ref.
Fixed 3D MEA	Probe	Microfabrication (planar multi-layer assembly); 3D printing (direct conductive ink printing or hollow-structure capillary filling)	Direct insertion into 3D tissue; depth-selective readout; post-culture readout platform	Invasive (tissue damage in dense/long-term models); single-shank or 1D-arranged multi-shank confined to the depth axis only; true 3D recording requires 2D (x-y)-arranged Michigan-probe type shank arrays	Neuron-hydrogel composites, brain slices, spheroids, organoids	[40, 46–60]
	Tip	3D printing (inkjet/aerosol-jet conductive ink printing; SLA printing with conductive coating)	Access to cells at intermediate depths; post-culture readout platform; compatible with existing planar MEA instrumentation	Larger uninsulated tip area increases the spatial extent of signal acquisition, lowering spatial resolution; high aspect ratio raises mechanical breakage risk	Neuron-hydrogel composites, brain slices, ex vivo tissue, organoids	[41, 61–71]
	Planar mesh	Microfabrication	Tissue fused/embedded within MEA during growth; records from interior neurons non-invasively	Confined to a single lateral (x-y) plane; does not constitute true 3D recording, as activity along the depth (z) axis cannot be independently resolved	Self-assembling/self-organizing models, organoids	[42, 72]
	3D mesh	3D printing (hollow insulating structure formed by DLP, then filled with conductive ink by capillary action); Microfabrication	Achieves true 3D recording; simultaneously interfacing/patterning the 3D tissue shape; records from interior neurons non-invasively	Suited only when tissue formation and device integration occur simultaneously	Neuron-hydrogel constructs, Self-assembling tissue models	[20, 73–75]
	Curved surface	Microfabrication with soft/elastic materials or 3D printing	Stable contact and positional stability with spherical tissue; suited as a post-culture readout platform; multi-well, high-throughput compatible	Surface-only recording (no interior access); hemispherical design covers only half of the model surface	Organoids, spheroids	[43, 76, 77]
Compliant 3D MEA	Embedded stretchable mesh	Microfabrication with soft/elastic materials (sub-micrometer substrate features)	Records from interior neurons non-invasively; Achieves true 3D recording	Embedded electrode positions drift during tissue growth; difficult fabrication to high throughput	Organoids	[44, 78–81]
	Surface- contacted stretchable mesh	Microfabrication with soft/elastic materials (sub-micrometer substrate features)	Post-culture readout platform enables long-term chronic monitoring; multi-organoid readout; Achieves true 3D recording	Surface-only recording (no interior access); difficult fabrication to high throughput	Organoids, assembloids	[82–85]
	Wrapping	Microfabrication with self-folding or mechanically guided bending process	High surface coverage from multiple directions; high electrode density via 2D fabrication; post-culture readout platform; Achieves true 3D recording	Surface-only recording (no interior access); the structure must be mechanically matched to each model’s size and shape to ensure stable contact	Spheroids, organoids, assembloids	[45, 86–92]

## Functional advances in 3D MEAs

In the field of *in vitro* 3D neuronal network model research, 3D MEA technology is rapidly advancing toward more precise signal interpretation through microelectrodes arranged in 3D space. This progress is driven not merely by increasing structural complexity, but by the need to more accurately analyze the physiological complexity of 3D neuronal network models. Electrophysiological analysis of these models has expanded beyond basic parameters such as spontaneous spike firing rate and burst rate, toward spatially resolved network analyses including network burst propagation [7, 20], spatially resolved synchronization [9, 20, 33, 40], oscillatory dynamics [33, 113], and functional connectivity [33, 40, 45, 92, 113], as well as evoked response analysis [20, 33, 113] within a single or modular 3D neuronal network model. As microelectrode distributions have expanded from the tissue surface to both the interior and exterior, the spatial origin and propagation of signals can be interpreted with greater precision [20]. These advances reflect a shift from simple activity measurements toward the characterization of spatial functional organization and circuit-level interactions within 3D neuronal networks. These trends can be categorized into four types.

First, 3D MEAs have advanced toward higher electrode density and greater spatial diversity. Early devices focused primarily on structural implementation, with relatively low electrode counts and simple electrode arrangements. More recently, advances in microfabrication, and high-resolution printing technologies have driven efforts to integrate more electrodes in three dimensions within a confined structure [45, 106]. This enables neural activity to be recorded with finer spatial resolution, contributing to the analysis of local circuit functions within 3D tissue models.

Second, the role of 3D MEAs has expanded beyond simple recording devices toward neuromodulation platforms. There is growing demand for multifunctional platforms that integrate electrical [20, 40], optical [40, 92, 100], and chemical [40, 53] stimulation alongside signal recording. Neuromodulation technologies allow selective depolarization or hyperpolarization of neurons at specific locations, enabling precise control of neural circuits within the network model. This has enabled a shift from spontaneous activity-based connectivity analysis toward evoked response-based connectivity analysis and stimulus propagation mapping, providing more precise characterization of functional network properties.

Third, 3D MEAs have evolved to incorporate structural design capabilities for 3D neuronal network models [20, 45]. The functional activity of a neural network is largely determined by its structural organization, a relationship that has been extensively studied in neuroscience [2, 114]. Building on this principle, 3D neuronal network models with defined structural patterns have been fabricated using platforms such as micro-molding [25–27, 45], bioprinting [31, 36, 37, 115, 116], and scaffolding [20, 117]. More recently, 3D MEAs with built-in patterning capabilities have been developed [20, 45]. These platforms use liquid-patterning technology-based scaffolds to define and maintain tissue geometry [118–120] while enabling interfacing with microelectrodes arranged in three dimensions to match the tissue structure. This enables structurally matched signal acquisition from complexly shaped 3D neuronal network models through a customized interfacing strategy optimized for each model geometry, ranging from modular cubic-unit structures to 3D doughnut configurations [20]. In addition, a wrapping-type 3D MEA incorporating micro-molding technology has been developed, enabling the formation of organoids into cubic, pyramidal, and asymmetrical shapes [45].

Fourth, there is growing interest in non-invasive 3D MEAs for interfacing with brain organoids. While probe-type or insertable electrodes offer advantages for recording signals from within the tissue, they can cause tissue damage during insertion and compromise long-term stability. As a result, mesh, wrapping, and stretchable mesh architectures that conformally wrap around the tissue surface or become naturally integrated during tissue growth have gained considerable attention. In particular, as organoids are typically cultured in large quantities over long periods, selective interfacing with specific models at desired time points is essential, driving active development of wrapping [45, 86, 92] and curved surface [43, 76, 77]-structured 3D MEAs suited for this purpose.

In summary, current advances in 3D MEA technology extend beyond simple 3D structural implementation, progressing along four key axes: high spatial resolution recording, multi-functionalization, customization of 3D tissue model structure, and non-invasive interfacing. These advances reflect the evolution of 3D MEAs from a simple arrangement of microelectrode into sophisticated bioelectronic interfaces capable of precisely recording and modulating the function of *in vitro* 3D neuronal network models.

## Limitations and perspective of 3D MEA technology

Although 3D MEA technology has emerged as a promising platform for electrophysiological recording from *in vitro* 3D neuronal network models, several structural and functional limitations remain, many of which arise from an unresolved trade-off among invasiveness, spatial resolution, and the stability of electrode distribution. This trade-off is particularly pronounced for 3D MEAs intended for organoid and spheroid models. As interest grows in organoids and spheroids cultured over long periods, there is increasing demand for 3D MEAs that can readily interface with these models without compromising their biological integrity. Conventional probe- and tip-type 3D MEAs achieve high spatial resolution by inserting electrodes directly into the tissue, but this invasive interfacing approach can induce localized cell death and reduce long-term culture stability [53–56]. Non-invasive approaches reduce this damage but introduce their own trade-offs. Embedded stretchable mesh architectures offer insufficient control over electrode distribution and result in irregular positioning relative to the 3D neuronal network structure [44, 78–81]. In contrast, surface-contacted stretchable mesh architectures [44, 78–81] and wrapping architectures [82–85] avoid insertion altogether but are primarily limited to surface signal recording, trading interior spatial resolution for reduced invasiveness. These trade-offs therefore suggest that future 3D MEAs for organoid and spheroid applications should be designed to simultaneously achieve sufficient non-invasiveness, spatial resolution capable of probing internal regions of the model, stable electrode distribution, and biocompatibility suitable for long-term culture periods.

As described above, 3D MEAs have evolved into diverse structural forms. However, because they still rely on microelectrodes to detect neuronal signals, their basic operating principles and recording performance requirements remain similar to those of conventional 2D MEAs. Low noise (~ 5 µV_RMS_) and low impedance at 1 kHz (typically several tens of kΩ) remain essential for high-signal-to-noise ratio (SNR) neural recording [121]. When the target shifts from a planar 2D network to a 3D neuronal network distributed throughout 3D space, additional limitations arise. Unlike 2D cultures, neurons are distributed across the z-axis, making close physical contact between neurons and microelectrodes more difficult to achieve [7, 9]. This reduces the number of active channels that can reliably detect neuronal signals in 3D neuronal network models. To address this limitation, recent studies have explored strategies such as pre-attaching neurons to microelectrodes prior to interfacing with 3D neuronal network models [7], or positioning the 3D model within the device such that tissue volume expansion during growth increases physical contact with embedded microelectrodes [45], thereby improving active channel yield. These findings highlight a growing emphasis on interfacing strategies that maximize active channel yield to achieve the spatial resolution necessary for network-level analysis.

In addition, existing 3D MEA technologies have not fully addressed the need for adaptive interfacing across diverse model geometries or for scalable platforms capable of high-throughput analysis with multiple models. Recent *in vitro* 3D neuronal network models are rapidly evolving into structurally complex forms, including various patterned models [20, 25, 26], and fused organoids [33–35], increasing the demand for 3D MEAs that can adaptively interface with a wide range of model geometries. Furthermore, given the large inter-model variability inherent to *in vitro* neuronal network models [122–124], high-throughput compatible platforms capable of processing multiple models simultaneously are essential for reliable quantitative analysis. Future 3D MEAs must therefore go beyond single-device performance improvements, toward platforms that meet these diverse requirements. Key goals include non-invasive interfacing, simultaneous recording from both the surface and interior of the tissue, precise electrode positioning, high electrode density, adaptability to different model shapes, and high-throughput analysis.

Beyond electrophysiological sensing with microelectrodes, 3D MEAs must incorporate neurochemical sensing and neuromodulation capabilities. Modern neuroscience research relies not only on electrical activity recording, but also on neurochemical signal monitoring [125] and diverse neuromodulation techniques such as neurochemical stimulation [126], optical-based technologies with optogenetic [127, 128] and photothermal stimulation [129]. In the 2D MEA field for *in vitro* neuronal network model analysis, multimodal sensing and stimulation technologies have already been actively applied. Systems combined with microfluidic channels enable drug delivery and local chemical stimulation [130, 131]. Light delivery to 2D MEAs has also been utilized for optogenetics-based precision stimulation and network modulation [132, 133]. In particular, photothermal stimulation using light-to-heat converting materials such as gold nanorods (GNRs) has demonstrated the potential for localized neuromodulation without genetic modification in *in vitro* neuronal network models [134–136]. MEAs integrated with temperature sensors have further been used to quantify the relationship between photothermal stimulation and neural activity [137, 138]. Future 3D MEAs for *in vitro* 3D neuronal network models are expected to evolve into highly functional platforms that enable the integration of multimodal neural sensing and stimulation throughout 3D space.

Furthermore, the development of next-generation 3D MEAs is closely tied to the growing interest in utilizing *in vitro* neuronal network models as biocomputing platforms [139, 140]. To practically exploit the unique computational properties of *in vitro* neuronal network models, including learning and memory mechanisms and high energy efficiency, interfaces capable of precisely reading and actively modulating the functional state of these models are essential [141]. Because biocomputing applications require a large number of inputs to support meaningful information processing, this necessitates the development of 3D MEAs incorporating a correspondingly large number of electrodes arranged across three dimensions. Such high-density 3D MEAs are needed not only to characterize spatially resolved functional networks in models such as organoids, but also to support biocomputing applications, with electrode counts approaching the very high densities exceeding 1,000 already demonstrated by CMOS-based high-density MEAs [139, 140]. Next-generation 3D MEAs are therefore expected to serve as a key enabling technology for exploring the information processing capabilities of *in vitro* 3D neuronal network models and advancing their potential as biocomputing platforms.
